# Genetic Background Influences Acute Response to TBI in Kindling-Susceptible, Kindling-Resistant, and Outbred Rats

**DOI:** 10.3389/fneur.2019.01286

**Published:** 2020-01-10

**Authors:** Robert J. Kotloski, Paul A. Rutecki, Thomas P. Sutula

**Affiliations:** ^1^Department of Neurology, University of Wisconsin School of Medicine and Public Health, Madison, WI, United States; ^2^Department of Neurology, William S. Middleton Memorial Veterans Hospital, Madison, WI, United States

**Keywords:** TBI, electroencephalography, BDNF, genetic background, rat

## Abstract

We hypothesized that the acute response to traumatic brain injury (TBI) shares mechanisms with brain plasticity in the kindling model. Utilizing two unique, complementary strains of inbred rats, selected to be either susceptible or resistant to seizure-induced plasticity evoked by kindling of the perforant path, we examined acute electrophysiological alterations and differences in brain-derived neurotrophic factor (BDNF) protein concentrations after a moderate-to-severe brain injury. At baseline, limited strain-dependent differences in acute electrophysiological activity were found, and no differences in BDNF. Following injury, pronounced strain-dependent differences in electrophysiologic activity were noted at 0.5 min. However, the divergence is transient, with diminished differences at 5 min after injury and no differences at 10 and 15 min after injury. Strain-specific differences in BDNF protein concentration were noted 4 h after injury. A simple risk score model generated by machine learning and based solely on post-injury electrophysiologic activity at the 0.5-min timepoint distinguished perforant path kindling susceptible (PPKS) rats from non-plasticity-susceptible strains. The findings demonstrate that genetic background which affects brain circuit plasticity also affects acute response to TBI. An improved understanding of the effect of genetic background on the cellular, molecular, and circuit plasticity mechanisms activated in response to TBI and their timecourse is key in developing much-needed novel therapeutic approaches.

## Introduction

Traumatic brain injury (TBI) is a major cause of death and disability, impacting all demographic groups. The mechanisms producing TBI are diverse, resulting in injuries ranging from mild to severe, with considerable variation in outcome. Prediction of sequela following TBI based on clinical presentation and imaging is challenging, as comparable injuries can have divergent outcomes, both at early and later stages. These observations suggest that other factors, such as genetic background, influence initial manifestations and secondary injury processes such as inflammation, lesion-induced plasticity and circuit repair, leading either to improvement or to delayed adverse consequences. Therefore, studying the role of genetic influences on the complex sequence of pathological and restorative processes that follow TBI may have important clinical implications.

Unsurprisingly, TBI acutely alters electrophysiologic activity. Human EEG studies obtained acutely after injury demonstrate primarily diffuse slowing (1–4). Animal studies, most conducted prior to the advent of modern recording and analysis techniques, demonstrate complicated results, likely resulting from differences in experimental approaches including experimental animals, mechanism of injury, and anesthesia. The majority of these studies demonstrate slowing and reduced amplitude of cerebral activities (5–9), with potentially epileptiform activity noted under some conditions (10, 11). Advances in recording capabilities, signal analysis, and improved methods for controlled and reproducible induction of experimental TBI offer an opportunity to advance understanding of acute changes in brain electrophysiologic activity after injury which, despite the importance of understanding brain injury at this early timepoint, has not been extensively explored.

Moderate-to-severe brain injuries involve direct mechanical damage with shearing forces, hemorrhage, excitotoxic necrosis, as well as more slowly evolving processes of plasticity which in a substantial subset of cases result in the delayed development of post-traumatic epilepsy (PTE) (12). Processes of circuit remodeling that increase susceptibility to seizures and include permanent structural and functional changes, such as the kindling model (13–16), may be relevant to the brain's response to TBI. For example, the neurotrophin brain-derived neurotrophic factor (BDNF) and its receptor tropomyosin receptor kinase B (TrkB) are critical for the progressive circuit alterations in the kindling model (17–19) and these same pathways play an important role following TBI (20, 21). Genetic differences have been demonstrated to be important in TBI, both in human (22–24) and animal studies (25), and many of these factors are also known to influence epilepsy. Therefore, genetic differences impacting plasticity in a model of epilepsy may be expected to impact response to TBI.

We hypothesized that the acute response to TBI shares mechanisms with brain plasticity in the kindling model, including involvement of BDNF. As the time course relevant for the development of TBI-related sequela such as PTE and cognitive deficits is unknown, we examined at the earliest time points for divergent responses to TBI in the inbred strains and outbred rats. In addition to the divergent responses to seizure-induction in the kindling model, these strains also demonstrate differences in behavior and learning paradigms which are known to change in brain injured animals (26–30). Therefore we examined acute electrophysiological alterations and BDNF expression after TBI in these unique, complementary strains as well as outbred SD rats.

## Materials and Methods

### Animals

We utilized novel strains of inbred Sprague-Dawley (SD) rats, selected for either increased rate (perforant path kindling susceptible, PPKS) or decreased rate (perforant path kindling resistant, PPKR) of perforant path kindling over the course of >15 generations (27). Additionally, out-bred SD rats, representing the parent strain, were acquired from a supplier (Envigo). Rats were 3–4 months of age at the time of surgery, and male and female rats were used in approximately equal numbers. Animals were maintained under 12 h light: 12 h dark cycles, with *ad libitum* food and water, in a vivarium under the care of the University of Wisconsin veterinarians. All animal handling and procedures were performed according to the NIH Guide for the Care and Use of the Laboratory Animals and the experiments were conducted under an approved protocol by the University of Wisconsin Institutional Animal Care and Use Committee.

### Surgery

**Figure 1:**
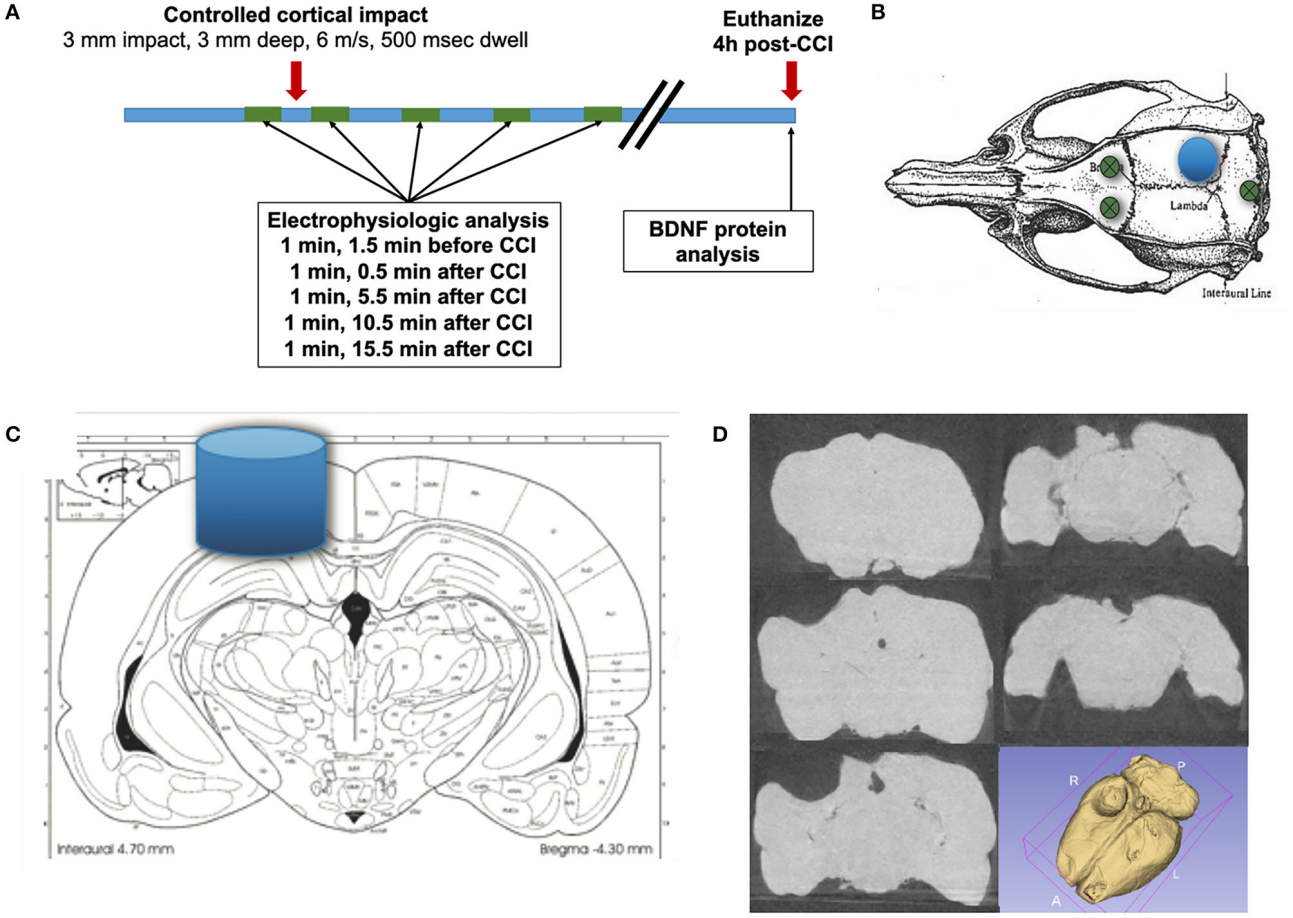
Experimental method. **(A)** Epidural recordings are performed prior to the CCI and continue for 20 min after the CCI. Rats are euthanized 4 h following CCI, and the brain is microdissected for protein analysis. **(B)** Electrical activity is recorded from bifrontal epidural electrodes, with a ground in the posterior skull (green circles). **(C)** A controlled cortical impact (CCI) with a 3 mm diameter blunt impactor is delivered over the right posterior cortex (blue circle and cylinder), with a depth of 3 mm, at 6 m/s, and with a dwell time of 500 ms. **(D)** A representative example of a lesion is demonstrated, with coronal CT slices and a 3D reconstruction (N.B. brains in this study were microdissected).

Prior to the procedure (Figure 1A), rats (PPKS *n* = 12, 7 males and 5 females; SD *n* = 8, 4 males and 4 females; PPKR *n* = 12, 8 males and 4 females) were weighed and anesthesia was induced with 5% isoflurane (Piramal) in 100% O_2_. The rat was placed into a stereotaxic frame with ear bars (Kopf Instruments) with bupivacaine (0.5%, SC, Fresenius Kabi USA, LLC) injected at contact points in the external auditory canals and along the midline of the scalp and with atropine (0.05 mg/kg IM, West Ward). Urethane (1.2 g/kg divided into three doses, IP, Sigma) was given immediately after induction with isoflurane, and isoflurane was weaned as tolerated, as assessed by tail flick in response to pinch and corneal reflex. Following the initial dosing, urethane-induced anesthesia persisted through the 4 h of this experiment. The scalp of the rat was shaved and prepared with topical betadine and alcohol along the midline. The skull was exposed and burr holes were drilled 1.5 mm anterior and 1.5 mm lateral (both left and right) to bregma, and a blind hole was drilled 1.5 mm posterior to lambda along the midline (Figure 1B). Coated stainless steel wire (0.010” bare diameter, 0.0130” coated, A-M Systems) was placed into these burr holes (into the epidural space for the anterior holes and into a blind hole in the skull for the posterior hole) and secured with a screw. A circular craniectomy, ~4 mm in diameter, was created over the right hemisphere, placed within the angle of the sagittal and lambdoid sutures (Figure 1B).

Isoflurane was completely stopped at least 10 min prior to recording electrical activity from the left and right epidural electrodes. Electrophysiologic recordings were performed utilizing an XLTEK EEG acquisition system (Neuroworks, version 7.1.1) with an EEG32U amplifier (sampled at 1,024 Hz). Electrophysiologic activity was recorded for 5 min prior to delivery of a CCI and for 20 min following injury (Figure 1A). CCI was delivered by Leica Impact One Stereotaxic Impactor (Leica), utilizing a 3 mm circular blunt impact tip with a velocity of 6 m/s and a dwell time of 500 ms (Figure 1C). As the brains of rats in this study were microdissected, a representative chronic injury, as visualized by coronal CT images and a 3D reconstruction (Figure 1D) is presented. The images are from a PPKS rat, 6 months after a CCI identical to the injury utilized in this study.

Four hours after CCI a subset of rats (PPKS *n* = 7, 5 males and 2 females; SD *n* = 5, 3 males and 2 females; PPKR *n* = 7, 4 males and 3 females) were euthanized by decapitation under deep isoflurane anesthesia. Following decapitation, the brain was rapidly dissected on ice to isolate posterior cortex (midline to rhinal sulcus, bilaterally), hippocampus (bilaterally), and cerebellum. Brain tissue was frozen in liquid nitrogen and stored at −80°C. A set of control rats (*n* = 5, 3 males and 2 females, for each strain) from each strain were euthanized with isoflurane and decapitated without prior surgery or CCI.

### Electrographic Analysis

The CCI was marked on the EEG recording in real-time and was confirmed by the electrical artifact of the impactor. A 60 s epoch of EEG ending at 0.5 min prior to CCI was selected for as a pre-injury baseline. Post-injury 60 s epochs beginning 0.5, 5, 10, and 15 min after CCI were selected for analysis (Figure 1A). The EEG samples were exported as a text file and imported into Matlab (R2017b, Mathworks). Electrophysiologic activity was bandpass filtered, using an equiripple filter and retaining frequencies between 0.5 and 32 Hz, binned at 0.5 Hz intervals. Power spectral density functions, a measure of power at different frequencies, are generated using a short-time Fourier transform with a Hamming window of 512 points and an overlap of 128 points. The post-CCI power spectral density was normalized to the baseline total power for each rat. Spectral entropy, a measure of complexity of the signal, was calculated by $H_{sp}=-\sum_{i=f_{l}}^{f_{h}}P_{i}\text{ log }{\text{P}}_{\text{i}}$ where *P* is the power density, *f*_*i*_ and *f*_*h*_ are the lower (0.5 Hz) and upper (32 Hz) frequency limits, and power is normalized (31). Magnitude-squared coherence, a measure of the similarity between two signals, was calculated by $C_{xy}\left(f\right)=\frac{|P_{xy}{|}^{2}}{P_{xx}P_{yy}}$ where *P*_*xx*_ and *P*_*yy*_ are the power spectral densities of *x* and y, respectively, and *P*_*xy*_ is the cross power spectral density of *x* and *y*, was calculated with a window of 512 points and an overlap of 128 points. Kurtosis, a measure of the frequency of outliers of a signal and often used as a measure of “sharpness” for electrographic activity, was calculated as the fourth standardized moment, $k=\;\frac{{E\left(x-\mu \right)}^{4}}{\sigma^{4}}$. Line length, often used as a measure of electrographic activity, was calculated by $l=\;\sum \sqrt{{\left(x_{i+1}-x_{i}\right)}^{2}+{\left(y_{i+1}-y_{i}\right)}^{2}}$.

### BDNF Protein Concentration

Dissected brain tissue (cortex, hippocampus, cerebellum) was collected, stored at −80°C, was thawed and homogenized by pestle in RIPA buffer (50 mM Tris-HCl, pH 7.5, 150 mM NaCl, 1% Triton X-100, 1% sodium deoxycholate, 0.1% SDS, 2 mM EDTA, Teknova) with protease inhibitor cocktail (104 mM AEBSF, 80 μM Aprotinin, 4 mM Bestatin, 1.4 mM E-64, 2 mM Leupeptin, and 1.5 mM Pepstatin A, Sigma). The homogenized tissue was left on ice for 15 min, and then centrifuged at 15,000 RCF for 15 min at 4°C. The supernatant was retained and its protein quantitated by a BSA protein assay (Pierce, ThermoFisher). The samples were acid treated with addition of HCl to a pH of 2–3 for 15 min, then neutralized with NaOH. BDNF content was assayed by a sandwich enzyme-linked immunosorbent assay (ELISA) (BDNF Emax ImmunoAssay System, Promega), utilizing a monoclonal anti-BDNF antibody for plate coating, and a human polyclonal anti-BDNF antibody with an anti-IgY HRP conjugate for colorimetric detection. A BDNF protein standard curve, performed in duplicate, was included on all plates. All samples were assayed in triplicate and then averaged. BDNF protein was quantified relative to total protein.

### Machine Learning Risk Score Model

A machine learning method, the Risk-Calibrated Supersparse Linear Integer Model (RiskSLIM) (32) uses optimization techniques to find the best logistic regression model, with bounded integer coefficients and a limited number of risk factors. The RiskSLIM method was utilized to generate a risk score for the rat belonging to the plasticity-susceptible strain (PPKS), as opposed to the non-plasticity-susceptible strains (SD or PPKR), based solely upon post-CCI electrographic parameters at 0.5 min after injury.

### Experimental Design and Statistical Analysis

Selection of samples of EEG data and BDNF ELISAs were performed in a blinded fashion. All results are presented as mean ± SEM, including power spectrum and magnitude-squared coherence. Data were analyzed by JMP Pro 13 (SAS Institute, Inc). Comparisons of power spectral density and interhemispheric coherence, using frequency bins from 0.5 to 32.5 Hz by 0.5 Hz steps, were analyzed by a Least Squares Fit model, and testing model construct effects for strain (PPKS vs. SD vs. PPKR), side (ipsilateral vs. contralateral), and/or timepoint (baseline vs. 0.5, 5, 10, or 15 min post-CCI). Otherwise data were analyzed by ANOVA, and using Tukey's HSD test for *post-hoc* analysis with α = 0.05. The groups included for each ANOVA are those presented on the corresponding figure. No differences were noted between males and females for any of the groups or experiments, and therefore the sexes were combined.

## Results

### Baseline

**Figure 2:**
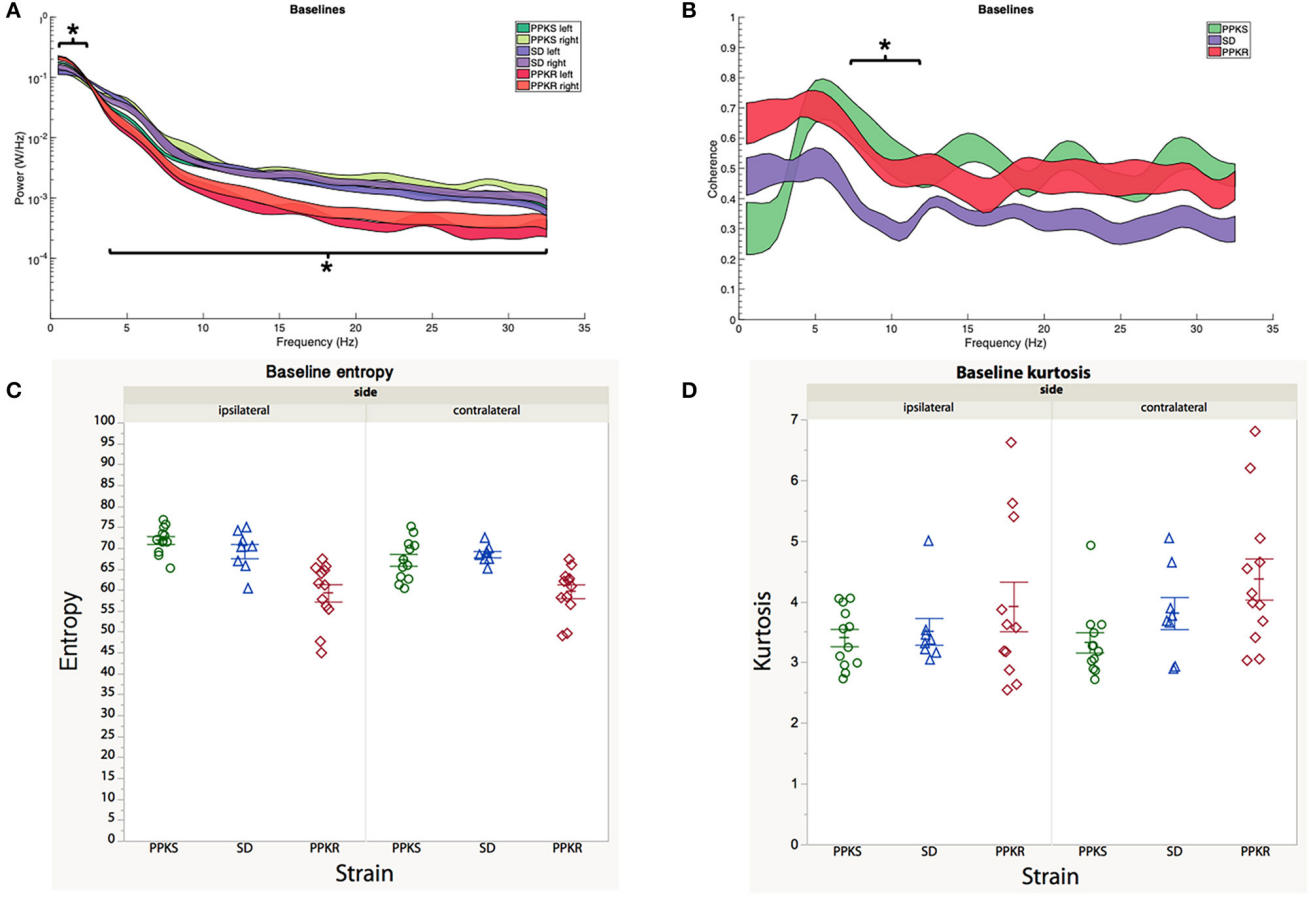
Baseline electrographic features. **(A)** The power spectrum (plotted as mean ± SE) of PPKR rats demonstrates increased power at 0.5–2 Hz and decreased power at 4–32 Hz, as compared to PPKS and SD rats (strain, LogWorth = 10.37, *p* < 0.01). **(B)** SD rats demonstrate decreased coherence between 6 and 11.5 Hz, as compared to PPKR and PPKS rats (strain, LogWorth = 3.06, *p* < 0.01). **(C)** No differences in entropy are noted among the strains prior to injury. **(D)** No differences in kurtosis are noted among the strains prior to injury. Significant frequency intervals marked by brackets, *p < 0.05.

At baseline, no differences in the power spectrum of the electrophysiologic activity were noted between the right (ipsilateral to the subsequent CCI) and left (contralateral to the subsequent CCI) hemispheres for any of the strains. Bilaterally, baseline electrophysiologic activity of PPKR rats demonstrated greater power in the slower frequencies (0.5 to 2 Hz) and less power at intermediate and faster frequencies (4 to 32 Hz), as compared to the baseline of SD and PPKS rats (Figure 2A). Magnitude-squared coherence at baseline demonstrated decreased coherence between the left and right hemispheres in SD rats at intermediate frequencies (6 to 11.5 Hz), as compared to PPKS and PPKS rats (Figure 2B). No differences were noted in either entropy (Figure 2C, Supplementary Table 1) or kurtosis (Figure 2D, Supplementary Table 1) at baseline. Prior to CCI, no differences were found in either total power or line length (Supplementary Table 1).

### Post-traumatic Changes in Electrophysiologic Activity

#### Immediate (0.5 min) Activity

**Figure 3:**
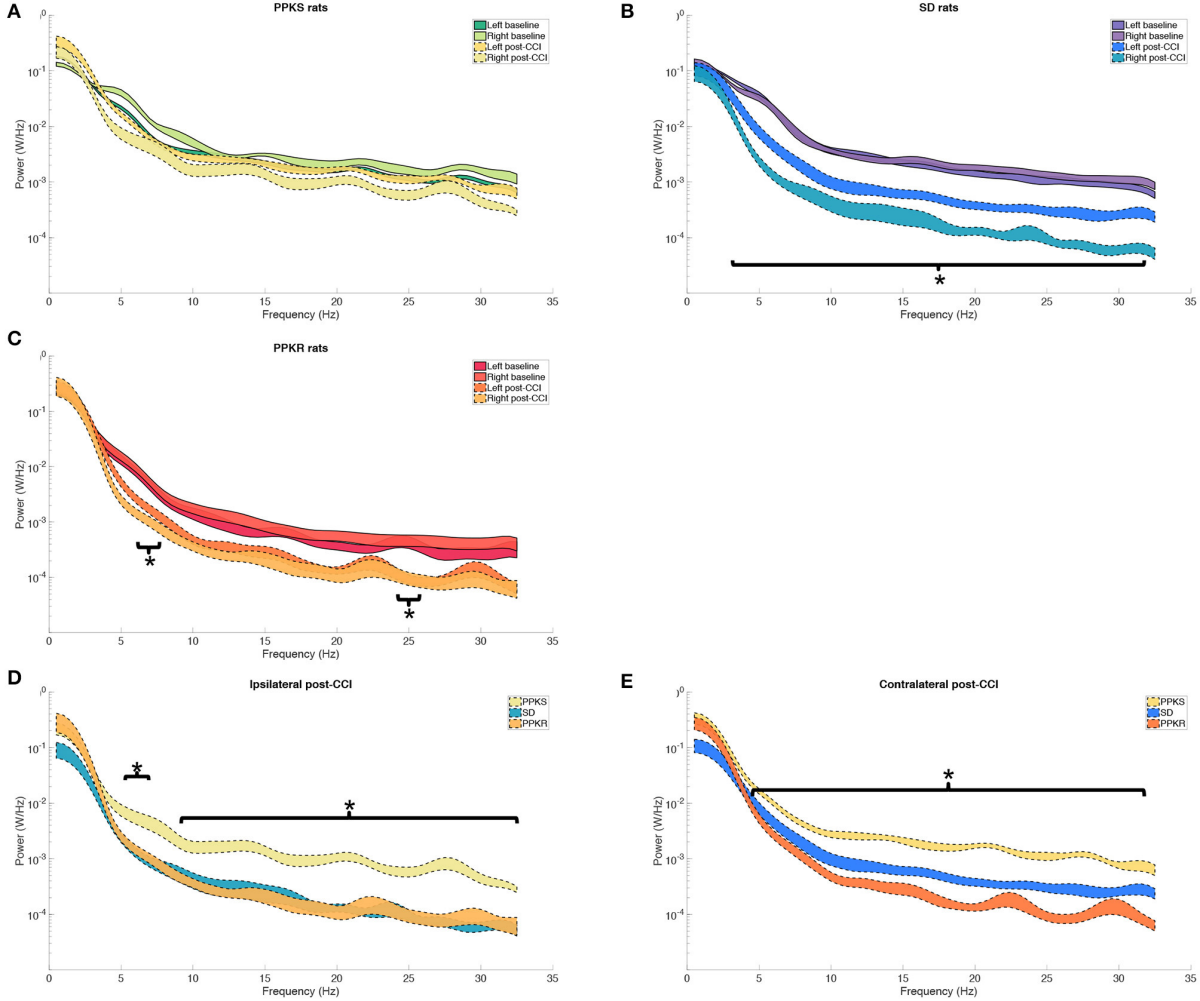
Pre- and post-CCI frequency distributions. **(A)** The power spectrum (plotted as mean ± SE) of PPKS rats demonstrates no changes in the frequency distribution following CCI. **(B)** SD rats demonstrate decreased power at 3.5–31.5 Hz after CCI, both ipsilateral and contralateral to the injury (timepoint, LogWorth = 8.40, *p* < 0.01). **(C)** PPKR rats demonstrate decreased power at 5.5–7 Hz and 24.5–25.5 Hz, both ipsilateral and contralateral to the injury (timepoint, LogWorth = 5.64, *p* < 0.01). **(D,E)** Following CCI, PPKR and SD rats demonstrate decreased power at faster frequencies, both ipsilateral (5.5–6.5 Hz, 9.5–32 Hz) (strain, LogWorth = 5.10, *p* < 0.01) and contralateral (4.5–31.5 Hz) (strain, LogWorth = 9.52, *p* < 0.01) to the injury, in comparison to PPKS rats. Significant frequency intervals marked by brackets, *p < 0.05.

At 0.5 min following CCI, PPKS rats did not demonstrate significant changes from baseline in the power spectrum, neither ipsilateral (right hemisphere) nor contralateral (left hemisphere) to the CCI (Figure 3A). The electrophysiologic activity of SD rats demonstrated a broad reduction in power both ipsilateral and contralateral to the CCI, with decreased power seen at 3.5 to 31.5 Hz, with a trend toward greater reduction in power ipsilateral to the injury (Figure 3B). PPKR rats also demonstrated bilateral reduction in power after CCI, albeit with statistically significant decreases limited to two narrow bands at 5.5 to 7 and 24.5 to 25.5 Hz (Figure 3C). Comparing across strains following CCI, broad reductions in the power of electrophysiologic activity were seen in SD and PPKR rats as compared to PPKS rats. Ipsilateral to the CCI, PPKS rats retained greater power at 5.5 to 6.5 and 9.5 to 32 Hz (Figure 3D), while contralateral to the CCI PPKS rats retained greater power at 4.5 to 31.5 Hz (Figure 3E).

**Figure 4:**
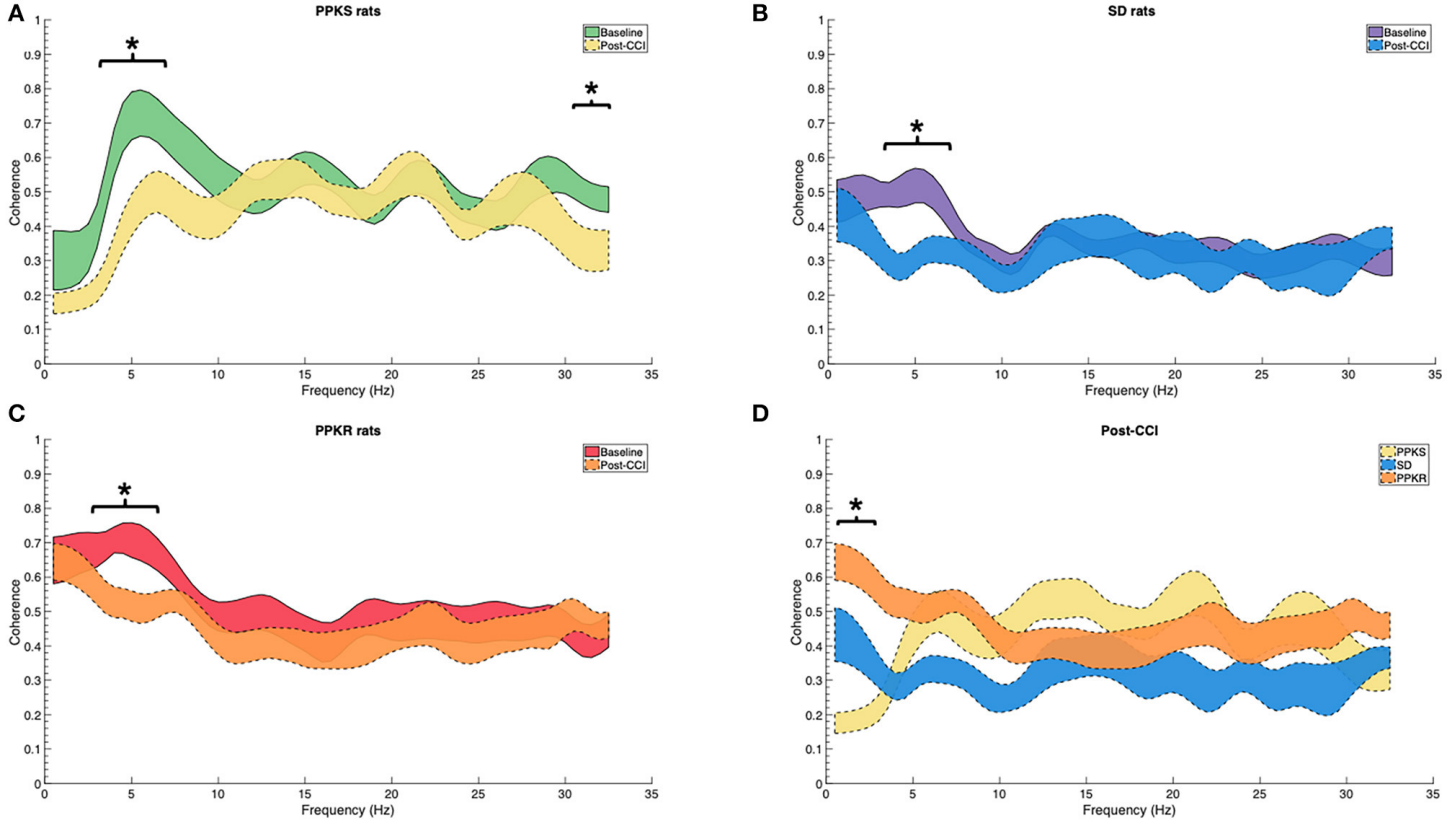
Pre- and post-CCI coherence. **(A–C)** Interhemispheric coherence decreases following CCI at intermediate frequencies in PPKS rats (3–7 Hz) (timepoint, LogWorth = 2.74, *p* < 0.01), SD rats (3–6 Hz) (timepoint, LogWorth = 2.63, *p* < 0.01), and PPKR rats (3.5–6.5 Hz) (timepoint, LogWorth = 2.34, *p* < 0.01). PPKS rats also demonstrate decreased coherence at 30.5–32 Hz. **(D)** Comparison of interhemispheric coherence after CCI demonstrates significant differences among all three strains at 0.5–2 Hz, with PPKS rats having lowest coherence, SD rats having intermediate coherence, and PPKR rats having highest coherence (strain, LogWorth = 7.03, *p* < 0.01). Significant frequency intervals marked by brackets, * *p* < 0.05.

At the 0.5-min post-injury timepoint, all strains displayed a loss of interhemispheric coherence in intermediate frequencies, with PPKS rats demonstrating a loss of coherence at 3 to 7 Hz (Figure 4A), SD rats demonstrating a loss at 3 to 6 Hz (Figure 4B), and PPKR rats demonstrating a loss at 3.5 to 6.5 Hz (Figure 4C). Comparing among strains, following CCI significant differences were seen in interhemispheric coherence between 0.5 to 2 Hz which differentiated all three strains, with PPKS rats having the lowest coherence, SD rats having intermediate coherence, and PPKR rats having the greatest coherence (Figure 4D). PPKS and SD rats demonstrated a decrease in entropy ipsilateral to the injury (**Figure 7A**, Supplementary Table 1), though not contralateral to the injury (Supplementary Table 1). PPKR rats did not demonstrate a change in entropy either ipsilateral (**Figure 7A**, Supplementary Table 1) or contralateral (Supplementary Table 1). No significant differences in entropy exist among post-CCI PPKS, post-CCI SD, baseline PPKR, and post-CCI PPKR rats (**Figure 7A**). Following CCI, kurtosis increased in PPKR rats ipsilateral to the injury (**Figure 7B**, Supplementary Table 1), though no change was seen contralateral to the injury (Supplementary Table 1). No differences in kurtosis were seen in PPKS or SD rats (Supplementary Table 1). Following CCI, no changes in total power or line length were found for PPKS, SD, or PPKR rats (Supplementary Table 1).

#### Early (5, 10, and 15 min) Activity

**Figure 5:**
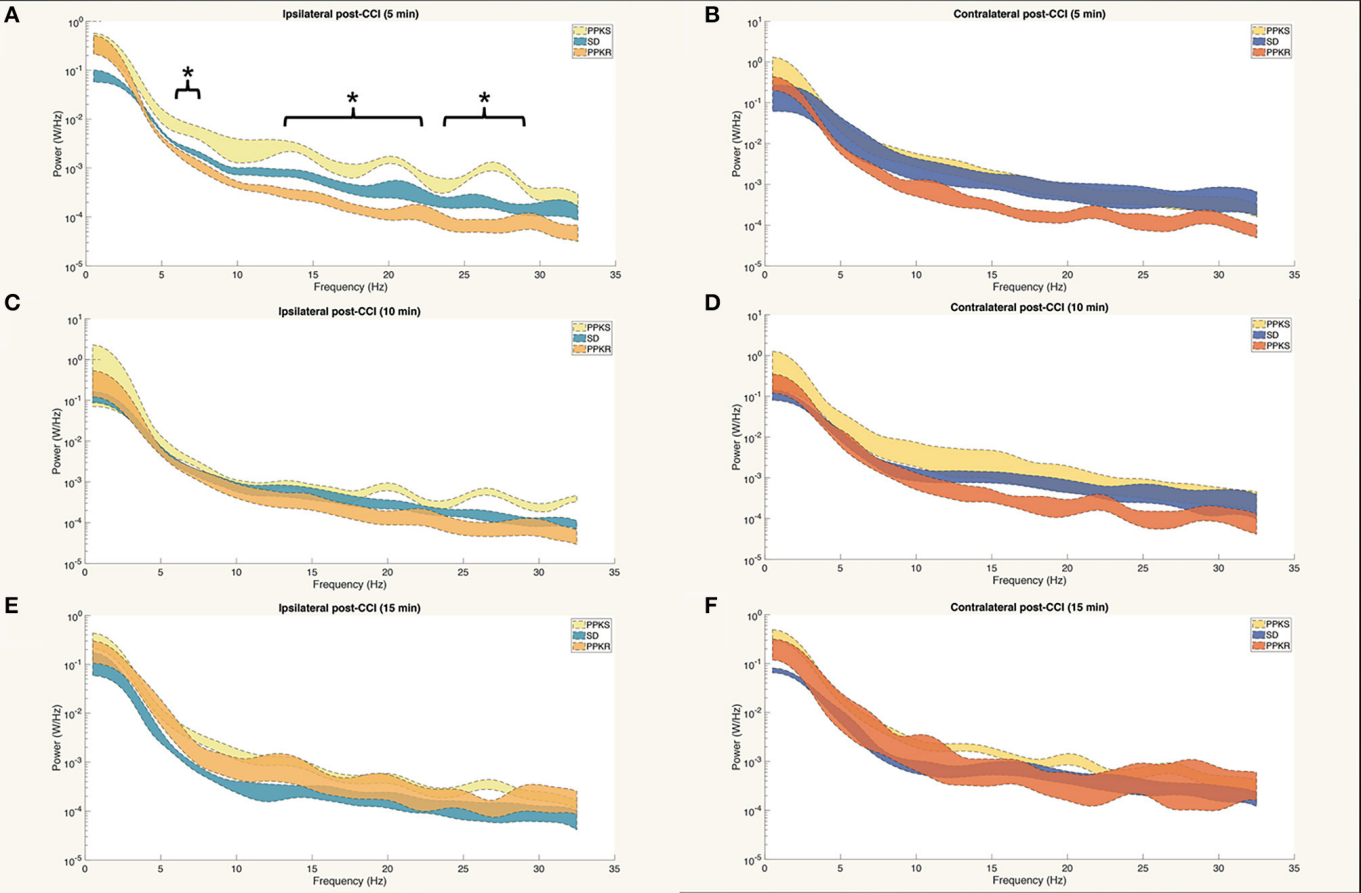
Power spectra at 5, 10, and 15 min after CCI. **(A)** At 5 min after CCI, the power spectrum of PPKS rats demonstrates greater power than PPKR rats at 6.5–7.5 Hz, 12.5–22 Hz, and 24–29.5 Hz ipsilateral to the injury (strain, LogWorth = 3.37, *p* < 0.01). **(B)** No statistically significant differences among strains are demonstrated in the power spectrum at 5 min after CCI contralateral to the injury. **(C–F)** No statistically significant differences among strains are demonstrated in the power spectra at 10 or 15 min after CCI, either ipsilateral or contralateral to the injury. Significant frequency intervals marked by brackets, *p < 0.05.

**Figure 6:**
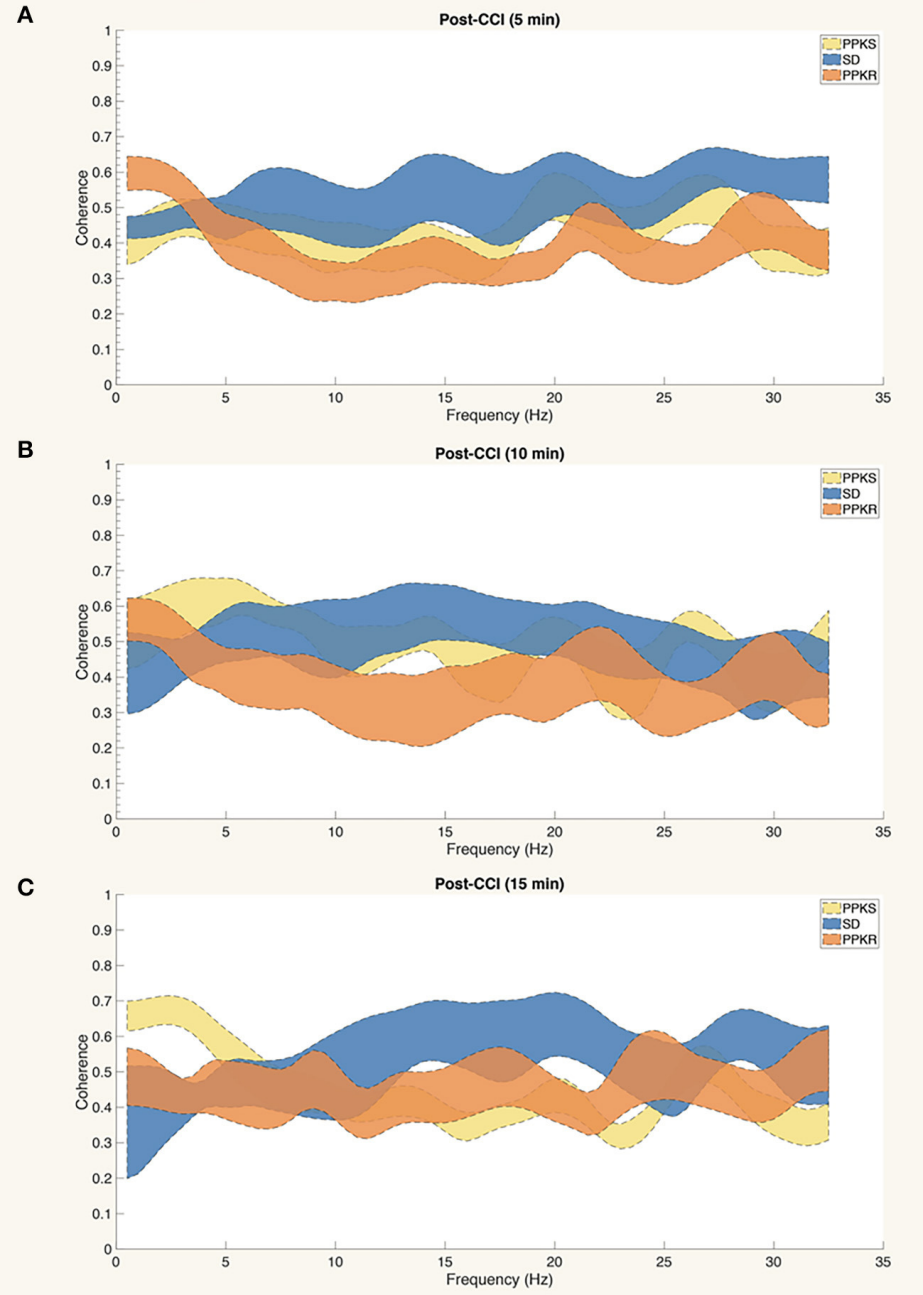
Interhemispheric coherence at 5, 10, and 15 min after CCI. **(A–C)** No statistically significant differences among strains are demonstrated in the interhemispheric coherence at 5, 10, or 15 min after CCI.

**Figure 7:**
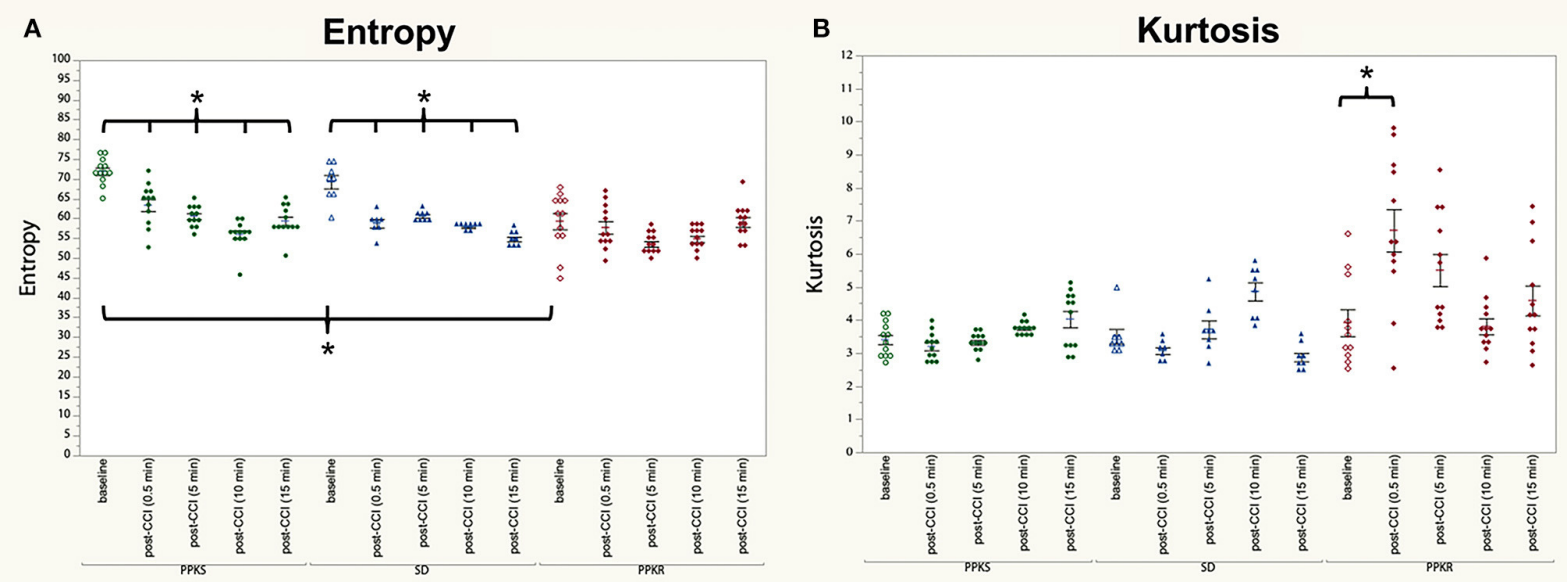
Pre- and post-CCI entropy and kurtosis. **(A)** PPKS and SD rats demonstrate a decrease in entropy ipsilateral to the injury following CCI, while PPKR rats do not demonstrate any change in entropy. **(B)** PPKS and SD rats do not demonstrate a change in kurtosis ipsilateral to the injury following CCI, while PPKR rats demonstrate an increase. * *p* < 0.05.

At 5 min following CCI, PPKS rats demonstrated greater power than PPKR rats at 6.5 to 7.5, 12.5 to 22, and 24 to 29.5 Hz ipsilateral to the injury (Figure 5A). SD rats did not demonstrate differences from the PPKS or PPKR rats ipsilateral to the injury at the 5-min timepoint. No inter-strain differences were noted at 5 min following CCI contralateral to the injury (Figure 5B), and no inter-strain differences were noted at 10 or 15 min following CCI, either ipsilateral or contralateral to the injury (Figures 5C–F). At the 5-, 10-, and 15-min timepoints no inter-strain differences in interhemispheric coherence were noted (Figures 6A–C). At 5, 10, and 15 min following CCI, PPKS and SD rats demonstrated a significant decrease in entropy ipsilateral to the injury (Figure 7A, Supplementary Table 1), but not contralateral to the injury (Supplementary Table 1). PPKR rats did not demonstrate a decrease in entropy at 5, 10, or 15 min after injury, either ipsilateral (Figure 7A, Supplementary Table 1) or contralateral to the injury (Supplementary Table 1). No statistically significant differences in kurtosis were noted at the 5-, 10-, or 15-min timepoints for the PPKS, SD, or PPKR strains, either ipsilateral or contralateral to the injury (Figure 7B, Supplementary Table 1). At 5, 10, and 15 min following CCI, no changes in total power or line length were found for PPKS, SD, or PPKR rats across time (Supplementary Table 1).

### BDNF Protein

**Figure 8:**
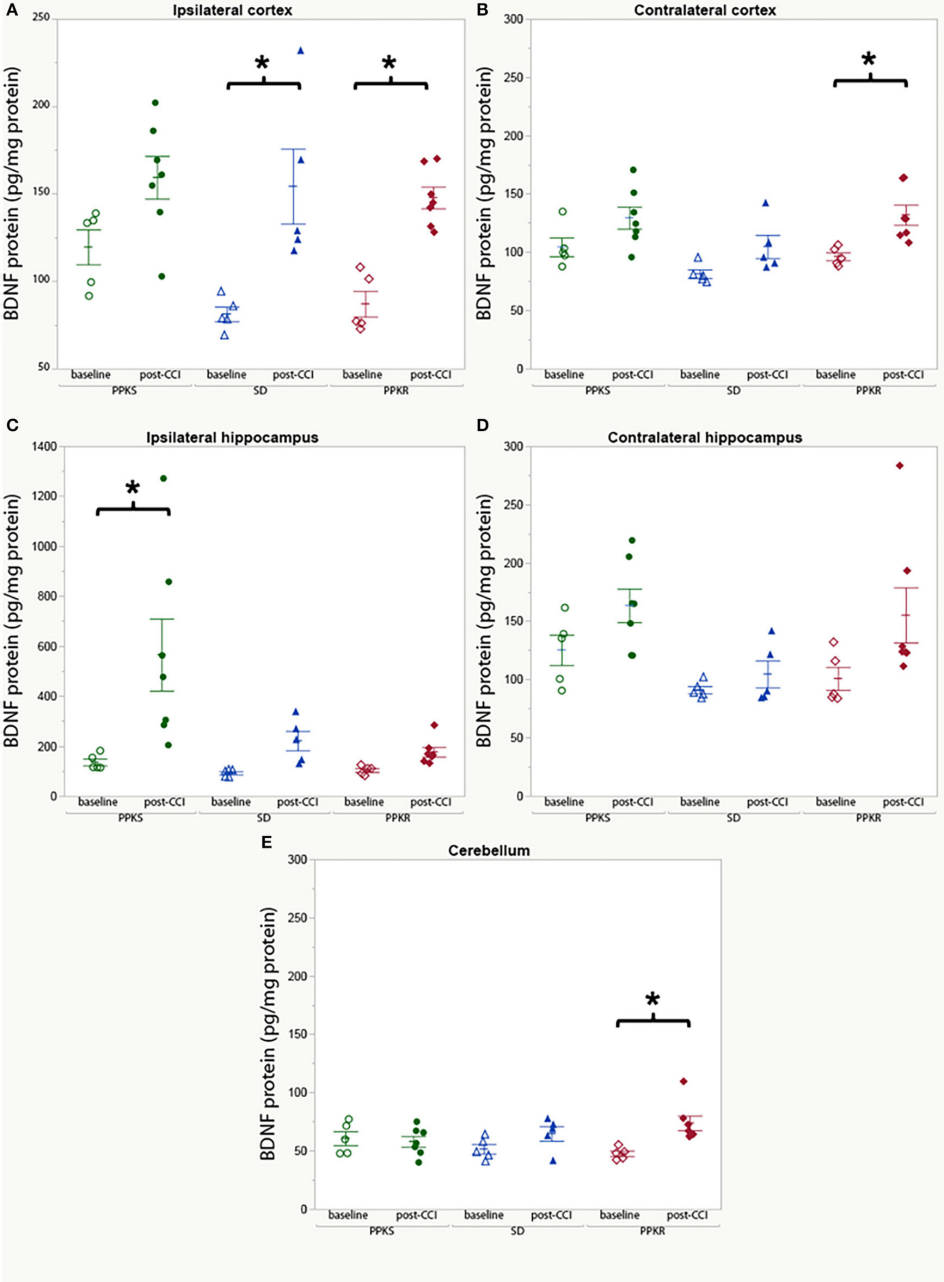
BDNF protein at baseline and following CCI. **(A)** No significant changes are noted in BDNF protein in cortex ipsilateral to the injury in PPKS rats, while SD and PPKR rats demonstrate an increase. **(B)** No significant changes are noted in BDNF protein in cortex contralateral to the injury in PPKS or SD rats, while PPKR rats demonstrate an increase. **(C)** PPKS rats demonstrate a large increase in BDNF protein in the hippocampus ipsilateral to the injury, while no significant differences are seen for SD or PPKR rats. **(D)** No significant differences are noted in BDNF protein in the hippocampus contralateral to the injury. **(E)** No change in BDNF protein in the cerebellum is noted in PPKS or SD rats, while PPKR rats demonstrate an increase in BDNF protein. *p < 0.05.

In uninjured rats, no differences in BDNF protein concentration were found among the strains in the ipsilateral cortex, contralateral cortex, ipsilateral hippocampus, contralateral hippocampus, or cerebellum (Supplementary Table 2). Comparing uninjured and injured rats, BDNF was greater in the cortex ipsilateral to the injury in SD and PPKR rats but no difference was seen in PPKS rats (Figure 8A, Supplementary Table 2). Similarly, BDNF was greater in the cortex contralateral to the injury in PPKR rats, but no difference was seen in PPKS or SD rats (Figure 8B, Supplementary Table 2). BDNF was greater in the hippocampus of injured PPKS rats than in uninjured PPKS rats, though no significant differences were noted in SD or PPKR rats (Figure 8C, Supplementary Table 2). No significant differences in BDNF were seen in the contralateral hippocampus, comparing uninjured and post-CCI rats from the PPKS, SD, or PPKR strains (Figure 8D, Supplementary Table 2). No differences in cerebellar BDNF were seen between uninjured and injured rats in the PPKS or SD strains, though cerebellar BDNF was greater in injured PPKR rats than in uninjured PPKR rats (Figure 8E, Supplementary Table 2).

### Risk Score Tool (RiskSLIM)

Rats were divided into two groups, either plasticity-susceptible rats (PPKS rats) or rats that are not plasticity-susceptible (SD and PPKR rats). Parameters of electrophysiologic activity recorded at 0.5 min after the CCI, including total (non-normalized) power; ipsilateral and contralateral percent band power in delta (0.5 to 4 Hz), theta (4.5 to 8 Hz), alpha (8.5 to 13 Hz), and beta (13.5 to 32.0 Hz); interhemispheric coherence in delta, theta, alpha, and beta bands; ipsilateral and contralateral entropy; ipsilateral and contralateral kurtosis; ipsilateral and contralateral line length, were collected. Dividing-point values for each parameter were identified with a partitioning approach based on the LogWorth statistic (JMP, SAS Institute Inc). The RiskSLIM method (32) was used, with a limit of 5 risk factors, integer coefficients of −1 to 1.

The resultant risk score tool incorporated one point for a magnitude-squared coherence in the delta band of <3, a beta band power of <3% over the contralateral hemisphere, and a kurtosis of <4 over the contralateral hemisphere (Supplementary Table 3). Using this tool, a score of 0 or 1 is associated with a 6.7% probability of the rat belonging to a plasticity-susceptible strain (PPKS), while a score of 2 is associated with a 75.0% probability and a score of 3 with an 88.9% probably of the rat belonging to a plasticity-susceptible strain (Supplementary Table 3).

## Discussion

We demonstrated that unique, complementary strains of inbred rats with a genetic background selected for susceptibility or resistance to kindling-induced plasticity exhibit distinct acute responses to moderate-to-severe TBI. Furthermore, the distinctive responses to TBI are brief, present at 0.5 min after injury, but are not seen at 5, 10, or 15 min after injury. Our findings reveal that important changes in electrophysiologic activity following brain injury. While these differences in electrophysiologic activity are transient, they are correlated with divergent patterns of BNDF protein expression, which is known to produce long-lasting and wide-ranging effects (33). Furthermore, these results provide an important foundation to explore later sequela of TBI in these unique strains.

These findings demonstrate the influence of genetic background affecting brain circuit plasticity on acute responses to TBI. The current experiments involve unique strains of inbred rats, selected for phenotype and therefore unbiased by expectations based on prior knowledge. Other investigations targeted at specific pathways have also demonstrated overlap between involving mechanisms of neuroplasticity and the response to TBI. In humans genetic polymorphisms in the BDNF gene are associated with differences in cognitive outcome after head trauma, both at early timepoints (1 month) (23) and at later timepoints (10–15 years) (24). Animal models of TBI have likewise demonstrated a connection between BNDF/TrkB signaling and TBI (21), including at times as early as 4 h after injury (20). Apolipoprotein E (ApoE) similarly has a role in neuroplasticity in normal physiology (34) and specific alleles of ApoE affect outcome after TBI (35–37). The genetic mechanisms associated with neuroplasticity in the kindling model and with response to TBI in the PPKS and PPKR strains is the subject of on-going investigations and has the potential to provide independent support for the role of BDNF/TrkB and other known mechanisms, as well as to identify unexpected or novel mechanisms.

The lack of extensive differences between the PPKS, PPKR, and SD strains at baseline is consistent with the selection method used for generating the inbred strains, which employed a response to a brain stimulus rather than a static trait. Therefore, the plasticity potential of the strains remains latent in the baseline state and few differences are noted. However, following injury more pronounced differences in electrophysiologic activity emerged between the strains at the 0.5-min timepoint, but these differences were much reduced at 5 min after injury and were no longer present at 10 or 15 min after the injury. Overall PPKR rats demonstrate a predominance of interhemispherically coherent slow frequencies and low signal complexity both at baseline and post-injury, which resembles the post-injury state of the PPKS and SD strains. Conversely, following injury PPKS rats have a power spectrum that resembles the uninjured, baseline state of SD rats. These findings are consistent with the hypothesis that the injury-induced electrophysiologic state in SD rats resembles the baseline state in PPKR rats, and that the injury-induced responses of outbred rats may not be fully present in PPKS rats.

In the uninjured state, examination of BDNF protein across the three strains demonstrated no significant differences. However, as with electrophysiologic activity, significant differences were noted when assessing the effect of injury. Injured PPKS rats demonstrated a large increase in hippocampal BDNF protein ipsilateral to the injury as compared to uninjured PPKS rats. Injured SD and PPKR rats demonstrated an increase in cortical BDNF as compared to uninjured controls which were not observed in the PPKS strain, with changes in cortical BDNF observed bilaterally in PPKR rats but only ipsilateral in SD rats. BDNF had been demonstrated to be involved in a plethora of brain processes, often with complicated anatomical and temporal patterns (38–40). Changes in BNDF have been described in multiple animal models of TBI (21, 41–45), and blood and CSF BDNF have been proposed as biomarkers for TBI (22, 46, 47), although the relationship between brain injury and BDNF appears complex. BDNF polymorphisms in humans are associated with differences in survival (48) and cognitive outcome after TBI (23, 24). Furthermore, BDNF is involved in multiple processes relevant to sequela of TBI, including neuroprotection (49), epileptogenesis (50), memory and cognition (51), and mental health conditions such as depression and post-traumatic stress disorder (PTSD) (52). The current findings identify anatomical patterns of BDNF very early following injury which are dependent on genetic background and which, given the divergent outcomes after TBI of the inbred strains, may be correlated with clinically important sequela of TBI. These results will help to advance our understanding of the intricate role of BDNF and associated signaling following injury, and to guide further development of emerging BDNF-related treatments for brain injury (53).

Risk models play an important role in medicine (54), informing prognosis and guiding treatment decisions. Currently our ability to predict outcome after TBI in clinical situations is limited, with most tools focused on survival (55, 56) or outcome at the level of the Glasgow Coma Scale (GCS) (57), rather than specific sequela, though some recent work suggests that EEG may be used to predict later PTE (58). We used a machine learning approach to generate a simple risk score model which distinguishes plasticity-susceptible rats (PPKS rats) from non-plasticity-susceptible strains (SD and PPKR rats) utilizing solely post-CCI electrophysiologic activity recorded at the 0.5-min timepoint. The ability to generate this model demonstrates the degree of divergence in the electrophysiologic response to TBI secondary to genetic background.

Several limitations regarding this study should be noted. As with essentially all animal models of TBI, the brain injury is produced under anesthesia and surgical conditions, neither of which are present in human TBI. Anesthesia likely has important effects on the brain injury (59) and on electrophysiologic activity. Urethane anesthesia was used in these experiments as it has a lesser impact on electrophysiologic activity than other agents (60). However, given the associated adverse effects of urethane, a survival surgery and subsequent follow-up to examine long-term outcome in these rats was not possible. Additionally, the electrophysiologic activity was recorded immediately after the injury, which would not be possible in clinical settings. This timepoint was chosen in an effort to identify the earliest point of divergence in response to injury among these strains, and this work succeeded in demonstrating the differences are apparent immediately (0.5 min) after injury, though the distinct patterns are no longer apparent at later timepoints in the early period (5, 10, or 15 min).

Our findings, including both measures of electrical brain activity and BDNF protein concentration, suggest a potential critical period for these conditions beginning immediately following injury. Future efforts will focus on the progression of these newly identified differences in the unique inbred strains beyond the acute timepoint and on direct correlation with sequela of TBI including PTE and cognitive and behavioral deficits. As electrophysiologic activity can be monitored non-invasively and relatively easily in humans, and as conditions such as PTE may be expected to have a robust signal in electrophysiologic activity, the ability to identify additional electrographic biomarkers in genetically-susceptible individuals is promising. Equally, molecules such as BDNF can be assayed in blood and CSF and may provide complementary prognostic information. An improved understanding of the cellular, molecular, and circuit plasticity mechanisms activated in response to TBI is key to developing much-needed novel therapeutic approaches. Given the large and growing burden of TBI, an improved understanding of the mechanisms leading to these conditions, including critical periods for their development and intervals during which disease-modifying intervention is possible, is vital for improved diagnosis and development of treatments.

## Data Availability Statement

The raw data supporting the conclusions of this article will be made available by the authors, without undue reservation, to any qualified researcher.

## Ethics Statement

The animal study was reviewed and approved by University of Wisconsin Institutional Animal Care and Use Committee.

## Author Contributions

RK designed and conducted experiments and wrote the manuscript. PR and TS assisted with experimental design and manuscript revisions. Inbred rat strains (PPKS and PPKR rats) were generated by TS.

### Conflict of Interest

The authors declare that the research was conducted in the absence of any commercial or financial relationships that could be construed as a potential conflict of interest.
