# Supplementary material for: Genetic Background Influences Acute Response to TBI in Kindling-Susceptible, Kindling-Resistant, and Outbred Rats
Source: Front Neurol. 2020 Jan 10;10:1286. doi: 10.3389/fneur.2019.01286 (PMC6968787; doi:10.3389/fneur.2019.01286)
Supplement: Supplementary file 1 [file Table_1.DOCX]

|  | **Baseline** | | | | | | **Statistical Significance** |  | **Post-CCI** | | | | | | | | **Statistical Significance** |
| --- | --- | --- | --- | --- | --- | --- | --- | --- | --- | --- | --- | --- | --- | --- | --- | --- | --- |
| **Electrophysiology** | **PPKS** | | **SD** | | **PPKR** | |  |  | **PPKS** | | | **SD** | | | **PPKR** | |  |
|  | **ipsi** | **contra** | **ipsi** | **contra** | **ipsi** | **contra** |  | **timepoint** | **ipsi** | **contra** | **ipsi** | | **contra** | **ipsi** | | **contra** |  |
| **Total Power (mW)** | 0.94 ± 0.21 | 1.15 ± 0.21 | 0.80 ± 0.08 | 1.78 ± 0.43 | 7.52 ± 2.92 | 6.68 ± 2.34 | **No differences at baseline** | **0.5 min** | 0.55 ± 0.12 | 1.36 ± 0.34 | 0.45 ± 0.08 | | 0.70 ± 0.10 | 4.65 ± 2.90 | | 8.17 ± 2.96 | **No differences from baseline** |
|  |  |  |  |  |  |  |  | **5 min** | 0.85 ± 0.48 | 1.53 ± 0.72 | 0.91 ± 0.17 | | 1.33 ± 0.82 | 4.45 ± 2.30 | | 4.97 ± 3.55 |  |
|  |  |  |  |  |  |  |  | **10 min** | 1.50 ± 0.42 | 1.54 ± 0.91 | 1.04 ± 0.51 | | 1.10 ± 0.89 | 2.56 ± 1.73 | | 3.56 ± 2.22 |  |
|  |  |  |  |  |  |  |  | **15 min** | 2.59 ± 0.69 | 2.10 ± 0.40 | 1.47 ± 0.90 | | 1.53 ± 0.92 | 1.36 ± 0.32 | | 1.42 ± 0.30 |  |
| **Line Length** | 0.102 ± 0.011 | 0.095 ± 0.008 | 0.084 ± 0.004 | 0.112 ± 0.015 | 0.095 ± 0.011 | 0.102 ± 0.012 | **No differences at baseline** | **0.5 min** | 0.052 ± 0.002 | 0.082 ± 0.009 | 0.032 ± 0.001 | | 0.058 ± 0.008 | 0.054 ± 0.014 | | 0.091 ± 0.015 | **No differences from baseline** |
|  |  |  |  |  |  |  |  | **5 min** | 0.052 ± 0.008 | 0.089 ± 0.008 | 0.052 ± 0.002 | | 0.083 ± 0.019 | 0.053 ± 0.013 | | 0.076 ± 0.015 |  |
|  |  |  |  |  |  |  |  | **10 min** | 0.059 ± 0.011 | 0.088 ± 0.007 | 0.047 ± 0.009 | | 0.070 ± 0.019 | 0.057 ± 0.013 | | 0.083 ± 0.016 |  |
|  |  |  |  |  |  |  |  | **15 min** | 0.067 ± 0.005 | 0.072 ± 0.002 | 0.038 ± 0.003 | | 0.60 ± 0.016 | 0.59 ± 0.007 | | 0.76 ± 0.010 |  |
| **Entropy** | **71.8 ± 0.9 *** | 67.1 ± 1.4 | **69.2 ± 1.7 *** | 68.5 ± 0.8 | **59.3 ± 2.1 *** | 59.6 ± 1.6 | **PPKR less than PPKS and SD at baseline (*p* < 0.01)** | **0.5 min** | **63.3 ± 1.5 *** | 63.5 ± 1.4 | **58.2 ± 1.1 *** | | 62.2 ± 1.0 | 57.6 ± 1.6 | | 56.6 ± 2.0 | **For PPKS and SD, ipsilateral decrease from baseline at 0.5, 5, 10, and 15 min ipsi**  **(*p* < 0.01)** |
|  |  |  |  |  |  |  |  | **5 min** | **60.6 ± 0.8*** | 58.5 ± 1.9 | **60.6 ± 0.5*** | | 63.0 ± 1.3 | 53.5 ± 0.7 | | 53.2 ± 1.8 |  |
|  |  |  |  |  |  |  |  | **10 min** | **55.9 ± 1.0 *** | 59.2 ± 2.1 | **57.8 ± 0.3 *** | | 63.8 ± 2.6 | 54.8 ± 0.8 | | 54.8 ±2.2 |  |
|  |  |  |  |  |  |  |  | **15 min** | **59.3 ± 1.1 *** | 58.5 ±1.1 | **54.8 ± 0.7 *** | | 61.9 ± 2.4 | 59.0 ± 1.5 | | 62.0 ± 2.2 |  |
| **Kurtosis** | 3.4 ± 0.1 | 3.3 ± 0.2 | 3.5 ± 0.3 | 3.8 ± 0.3 | 5.1 ± 1.2 | 4.4 ± 0.3 | **No differences at baseline** | **0.5 min** | 3.2 ± 0.2 | 4.0 ± 0.7 | 3.0 ± 0.1 | | 3.4 ± 0.2 | **6.7 ±  0.6 *** | | 6.0 ± 0.9 | **At 0.5 min for PPKR, ipsilateral increase from baseline (*p* < 0.01)** |
|  |  |  |  |  |  |  |  | **5 min** | 3.3 ± 0.1 | 3.0 ± 0.2 | 3.7 ± 0.3 | | 3.2 ± 0.2 | 5.5 ± 0.5 | | 5.3 ± 1.0 |  |
|  |  |  |  |  |  |  |  | **10 min** | 3.7 ± 0.1 | 3.7 ± 0.3 | 4.8 ± 0.3 | | 5.8 ± 0.8 | 3.8 ± 0.3 | | 3.8 ± 0.8 |  |
|  |  |  |  |  |  |  |  | **15 min** | 4.0 ± 0.3 | 4.5 ± 1.0 | 2.9 ± 0.2 | | 2.7 ± 0.1 | 4.5 ± 0.5 | | 6.0 ± 1.7 |  |

**Supplementary Table 1. Summary of electrophysiologic characteristics.** Statistical analysis was performed ANOVA for each strain (PPKS, SD, PPKR) comparing the baseline state to the post-CCI condition. For entropy interactions were found for strain*timepoint (F_8,256_ = 7.72), strain*side (F_2,256_ = 5.45), and timepoint*side (F_4,256_ = 2.93). For kurtosis a main effect was found for strain (F_2,256_ = 19.14) and an interaction for strain*timepoint (F_8, 256_ = 5.03). * indicates *p* < 0.05 by post-hoc analysis with Tukey’s HSD test.
