# Supplementary material for: Genetic Background Influences Acute Response to TBI in Kindling-Susceptible, Kindling-Resistant, and Outbred Rats
Source: Front Neurol. 2020 Jan 10;10:1286. doi: 10.3389/fneur.2019.01286 (PMC6968787; doi:10.3389/fneur.2019.01286)
Supplement: Supplementary file 2 [file Table_2.DOCX]

| **BDNF (pg/mg total protein)** | **Uninjured** | | | | | | | **Statistical Significance** | **4 hours post-CCI** | | | | | | **Statistical Significance** |
| --- | --- | --- | --- | --- | --- | --- | --- | --- | --- | --- | --- | --- | --- | --- | --- |
|  | **PPKS** | | **SD** | | **PPKR** | | |  | **PPKS** | | **SD** | | **PPKR** | |  |
|  | **ipsi** | **contra** | **ipsi** | **contra** | **ipsi** | **contra** | |  | **ipsi** | **contra** | **ipsi** | **contra** | **ipsi** | **contra** |  |
| **Cortex** | 119 ± 10 | 104 ± 8 | 81 ± 4 | 81 ± 4 | 87 ± 7 | 96 ± 3 | | **No differences at baseline** | 159 ± 12 | 129 ± 9 | **154 ±**  **21 *** | 104 ± 10 | **148 ± 6 *** | **132 ± 9 *** | **For SD, ipsilateral increase from baseline. For PPKR, bilateral increase from baseline  contra (*p* < 0.001) and ipsi (*p* < 0.001)** |
| **Hippocampus** | 136 ± 14 | 125 ± 13 | 92 ± 6 | 91 ± 3 | 103 ± 8 | | 101 ± 10 | **No differences at baseline** | **565 ±**  **144 *** | 163 ± 14 | 221 ± 39 | 104 ± 12 | 177 ± 19 | 155 ± 19 | **For PPKS, ipsilateral increase from baseline  ipsi (*p* < 0.001)** |
| **Cerebellum** | 61 ± 6 | | 52 ± 4 | | 48 ± 2 | | | **No differences at baseline** | 58 ± 4 | | 65 ± 6 | | **74 ± 6 *** | | **For PPKR, increase from baseline *(p* = 0.017)** |

**Supplemental Table 2. Summary of BDNF protein concentrations.** Statistical analysis was performed ANOVA for each strain (PPKS, SD, PPKR) comparing the uninjured state to the post-CCI condition. For the ipsilateral cortex an interaction was found for strain*injury (F_2,28_ = 4.09). For the contralateral cortex main effects were found for strain (F_2,28_ = 4.70) and injury (F_1,28_ = 17.04). For the ipsilateral hippocampus main effects were found for strain (F_2,28_ = 4.77) and injury (F_1,28_ = 11.21). For the cerebellum an interaction was found for strain*injury (F_2,28_ = 3.82). * indicates *p* < 0.05 by post-hoc analysis with Tukey’s HSD test.
