# Supplementary material for: Genetic Background Influences Acute Response to TBI in Kindling-Susceptible, Kindling-Resistant, and Outbred Rats
Source: Front Neurol. 2020 Jan 10;10:1286. doi: 10.3389/fneur.2019.01286 (PMC6968787; doi:10.3389/fneur.2019.01286)
Supplement: Supplementary file 3 [file Table_3.docx]

| **Risk of plasticity-susceptible genotype** | | | | | | | |  |
| --- | --- | --- | --- | --- | --- | --- | --- | --- |
| 1 . Magnitude-squared coherence in delta band < 3 | | | + 1 point | | . . . . . . | |  |  |
| 2 . Contralateral beta power percentage > 3% | | | + 1 point | | + . . . . . . | |  |  |
| 3 . Contralateral kurtosis < 4 | | | + 1 point | | + . . . . . . | |  |  |
| ADD POINTS FROM ROWS 1-3 | | | Score | | = . . . . . . | |  |  |
|  | | | | | |  | | |
| SCORE | 0 -1 | 2 | | 3 | | | |  |
| ACTUAL RISK | 6.7% | 75.0% | | 88.9% | | | |  |

**Supplementary Table 3.** **Risk scoring algorithm.** A simple risk scoring tool was constructed using a machine learning approach. Using only characteristics of the electrographic recording at 0.5 min following injury, the risk of being a member of the plasticity-susceptible (PPKS) strain can be predicted.
